# Anti-neutrophil Cytoplasmic Antibody (ANCA)-Negative Pauci-Immune Crescentic Glomerulonephritis: A Case Series From a Tertiary Care Hospital in South India

**DOI:** 10.7759/cureus.89817

**Published:** 2025-08-11

**Authors:** Anbalagan Suyambulingam, Arun K, V Jayakaran, Abinaya Venkatesan, Ebby Perin Mathisha

**Affiliations:** 1 General Medicine, Sree Balaji Medical College and Hospital, Chennai, IND

**Keywords:** anca-negative vasculitis, crescentic gn, immunosuppression, pauci-immune glomerulonephritis, picgn, rapidly progressive glomerulonephritis, renal biopsy, rpgn

## Abstract

Background: Pauci-immune crescentic glomerulonephritis (PICGN) is a critical form of renal disease characterized by a swift deterioration in kidney function and crescent formation in glomeruli, usually in the absence of notable immune deposits. While commonly associated with anti-neutrophil cytoplasmic antibodies (ANCAs), a subset of patients presents without detectable ANCA, complicating both diagnosis and management.

Objective: This case series presents an overview of the clinical manifestations, laboratory parameters, biopsy findings, and therapeutic outcomes observed in individuals diagnosed with ANCA-negative PICGN through histological confirmation.

Methods: This series reviews seven retrospective cases of ANCA-negative PICGN managed at a tertiary hospital in South India, focusing on clinical presentation, investigations, renal biopsy findings, treatment strategies, and outcomes.

Results: All seven patients exhibited acute kidney injury (AKI), significant proteinuria, and active urinary sediment. Inflammatory markers were elevated in all cases. Renal biopsies demonstrated 70-90% crescent formation with the absence of immune complex deposits. Serologic tests for ANCA, antinuclear antibody (ANA), and double-stranded DNA (dsDNA) were negative; complement levels remained within the normal limits. Three patients had identifiable urinary tract infections with Gram-negative organisms. Two patients developed pulmonary involvement. Each patient was treated with a combination of corticosteroids and cyclophosphamide, alongside supportive renal replacement therapy through dialysis. Four patients improved partially, one showed a slow response, while two succumbed during hospitalization.

Conclusion: ANCA-negative PICGN represents a severe crescentic glomerulonephritis with significant diagnostic and therapeutic implications. Prompt renal biopsy and early immunosuppressive therapy are essential for renal salvage. Despite treatment, outcomes remain variable, emphasizing the need for heightened clinical suspicion and timely intervention. ANCA-negative PICGN is an aggressive glomerular disease. Renal biopsy is indispensable in seronegative cases of rapid renal deterioration. Due to its aggressive nature, this form of glomerular disease requires timely histopathological evaluation and the immediate initiation of immunosuppressive therapy, especially when autoimmune serology is negative. Although partial recovery is possible, mortality remains a concern.

## Introduction

Pauci-immune crescentic glomerulonephritis (PICGN) is a severe renal condition marked by rapidly declining kidney function and crescent formation within the glomeruli, generally occurring in the absence of significant immune complex deposition on histopathological examination [1]. This condition is frequently associated with anti-neutrophil cytoplasmic antibodies (ANCAs), which serve as serological markers in most cases and play a role in pathogenesis through neutrophil-mediated endothelial injury [2].

Nevertheless, an estimated 10% to 20% of individuals diagnosed with PICGN based on biopsy findings do not exhibit detectable ANCA in their serum [3-5]. These individuals lack the serologic markers commonly used to detect small-vessel vasculitis, posing a diagnostic challenge. Clinically, ANCA-negative patients may present with hematuria, proteinuria, hypertension, and rapidly deteriorating renal function, similar to ANCA-positive cases [4]. The absence of ANCA can delay diagnosis and treatment, potentially contributing to worse outcomes [5].

The underlying immune-pathogenesis of ANCA-negative PICGN remains unclear. Hypotheses include localized immune activation, undetectable or novel antibodies, or complement-independent mechanisms contributing to crescent formation [6,7]. The histological findings, however, remain consistent, extensive crescents with minimal immune deposits [5].

Prognostically, several studies suggest ANCA-negative PICGN may follow a more severe course, with delayed renal recovery and increased likelihood of dialysis dependence, even when treated with standard immunosuppressive regimens [8,9]. Treatment primarily involves high-dose corticosteroids and cytotoxic agents, such as cyclophosphamide or rituximab, although the response is variable [8,10].

Given the rarity and heterogeneity of ANCA-negative cases, larger prospective studies are lacking, making individual case series essential in enhancing clinical awareness, guiding therapeutic strategies, and identifying prognostic patterns [7,8].

Furthermore, standardized assessment of kidney biopsy specimens is essential to ensure consistent interpretation of chronic damage, which may influence long-term renal outcomes and therapeutic decisions [11]. Histological evaluation methods developed for conditions like lupus nephritis, including assessments of crescent structure and chronic damage, provide valuable perspectives on disease evolution and may serve as a reference for grading lesions in ANCA-negative PICGN [12].

## Materials and methods

This retrospective observational study was conducted in the Department of General Medicine at Sree Balaji Medical College and Hospital, Chennai, Tamil Nadu, India, a tertiary care referral center serving a diverse patient population across South India. The study was approved by the Institutional Human Ethics Committee (009/SBMCH/IHEC/2025/1995), and written informed consent was obtained from all patients or their legal representatives.

The study included seven patients who were admitted between January 2022 and March 2024 with clinical features suggestive of rapidly progressive glomerulonephritis and subsequently diagnosed with PICGN in the absence of ANCA positivity. To maintain diagnostic precision, only cases confirmed through renal biopsy were considered for inclusion. Patients with lupus nephritis, IgA nephropathy, anti-glomerular basement membrane (anti-GBM) disease, or other immune-complex-mediated glomerulonephritis were excluded. Those who had incomplete records were excluded, and we also excluded the cases if serological testing was not performed.

Data collection

A comprehensive retrospective analysis was conducted using hospital medical records of patients diagnosed with ANCA-negative PICGN. The data extraction process encompassed a wide range of clinical and investigative parameters. Demographic details such as age and sex were recorded, alongside presenting symptoms including generalized fatigue, lower limb edema, reduced urine output, breathlessness, joint pain, and muscle aches. Vital signs, particularly blood pressure on admission, were noted to evaluate any hypertensive emergencies or fluctuations during hospitalization.

Laboratory assessments included a complete hemogram to determine hemoglobin levels, total leukocyte count, and platelet count. Renal function was evaluated through serum creatinine and blood urea levels, while serum electrolytes, specifically sodium, potassium, and bicarbonate, were assessed to detect metabolic imbalances. Inflammatory activity was gauged using erythrocyte sedimentation rate (ESR) and C-reactive protein (CRP) levels. Urinalysis findings were documented, including the degree of proteinuria, hematuria, and the presence of urinary casts or dysmorphic red blood cells.

Further serological testing was performed to rule out autoimmune etiologies. This included antinuclear antibody (ANA), anti-double-stranded DNA (anti-dsDNA), and anti-GBM antibodies, along with complement components C3 and C4. Both cytoplasmic (c-ANCA) and perinuclear (p-ANCA) were tested in all patients. To identify concurrent infections, urine cultures were obtained to detect common Gram-negative uropathogens. Radiologic evaluation, including chest X-rays and high-resolution computed tomography (HRCT) scans, was used to assess for pulmonary involvement such as infiltrates or cavitary lesions. Histopathological examination of renal biopsy specimens was undertaken using light microscopy and periodic acid-Schiff (PAS) staining, with immunofluorescence employed to confirm the extent of crescent formation and exclude significant immune complex deposition.

Treatment protocol

All individuals received treatment based on the institutional protocol for crescentic glomerulonephritis. This included immunosuppressive management initiated with intravenous methylprednisolone at a dose of 1 gram daily for three consecutive days, followed by maintenance therapy with oral prednisolone at 1 mg/kg/day. Cyclophosphamide was initiated either orally (2 mg/kg/day) or intravenously (0.5-0.75 g/m² monthly pulses), based on renal function and clinical status. Adjunctive treatments included antihypertensives, diuretics, antibiotics when indicated, and correction of electrolyte imbalances.

Patients with oliguria or worsening renal function were initiated on hemodialysis, with the number of sessions recorded. All patients were closely monitored for signs of infection, response to therapy, and complications during hospitalization.

Outcome assessment

The principal outcomes evaluated in this case series included the degree of renal function recovery, the requirement for continued dialysis, and in-hospital mortality. Renal improvement was primarily assessed through serial measurements of serum creatinine levels. Based on the clinical course and biochemical trends, outcomes were classified into three distinct categories. The first, "partial recovery," was defined as a measurable improvement in renal parameters accompanied by independence from dialysis at the time of discharge. The second, labeled "slow response," referred to a gradual but incomplete improvement in renal function, often characterized by persistent elevation in serum creatinine levels despite treatment. The third outcome, "death," denoted patients who succumbed during hospitalization, regardless of their renal status at the time of death.

Given the limited sample size of seven patients, a purely descriptive analytical approach was employed. Statistical inferences were not attempted due to the heterogeneity and small cohort. Instead, emphasis was placed on detailed clinical documentation of each case. The results were structured as a narrative case series to illustrate the variation in presentation, histopathological features, and response to immunosuppressive therapy.

## Results

A total of seven patients with biopsy-confirmed ANCA-negative PICGN were included in this series. All patients presented with acute kidney injury (AKI) characterized by elevated serum creatinine, uremia, and active urinary sediment. The cohort included four males and three females, with a mean age of 55 years (range: 43-63 years).

Baseline clinical features

**Figure 1:**
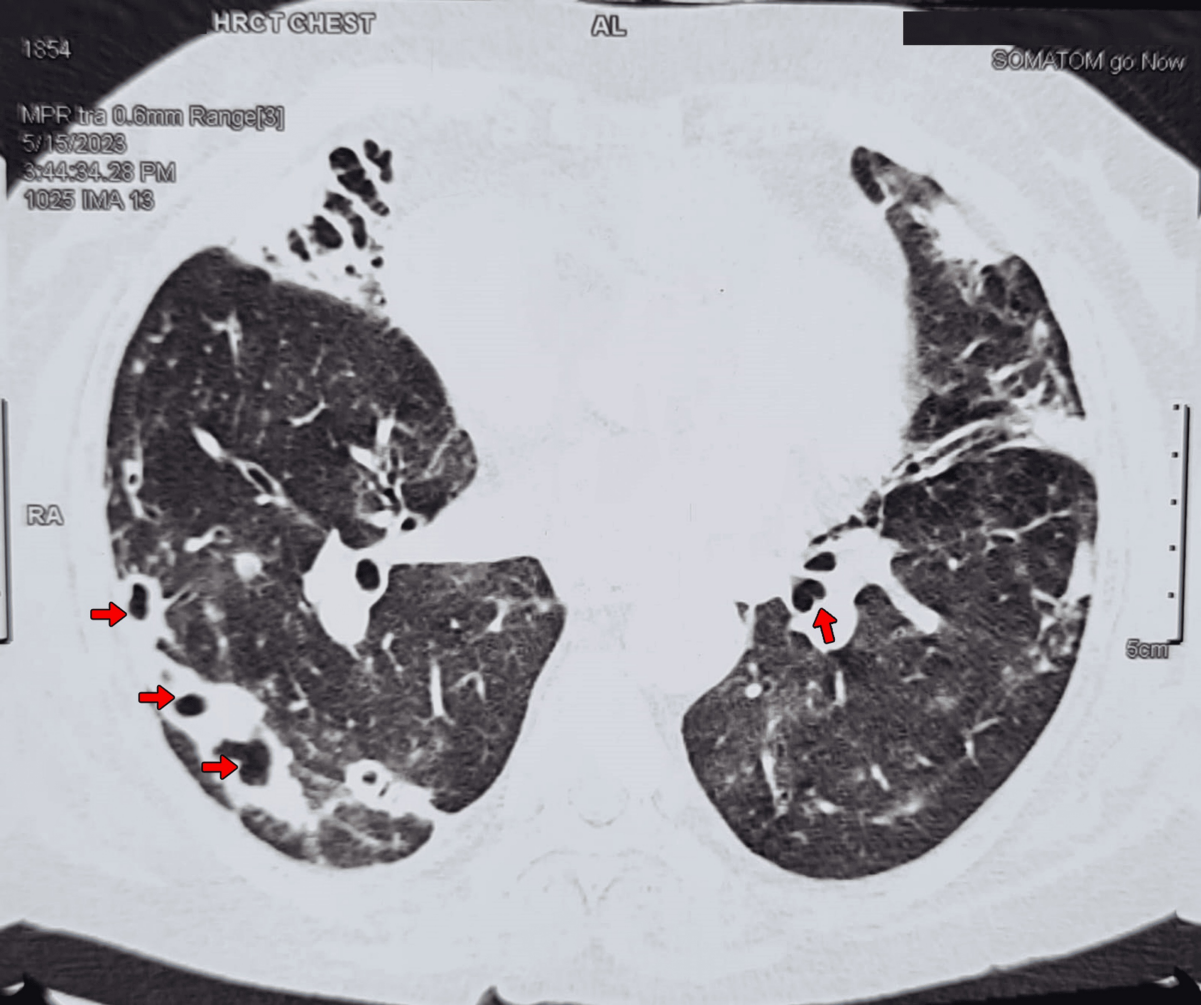
HRCT chest (axial view) – bilateral pulmonary cavities High-resolution computed tomography (HRCT) chest (axial plane) demonstrating multiple thick-walled cavitary lesions in both lungs (red arrows). These findings raised the differential diagnosis of granulomatosis with polyangiitis versus secondary infection in the context of ANCA-negative vasculitis. ANCA: anti-neutrophil cytoplasmic antibody

**Figure 2:**
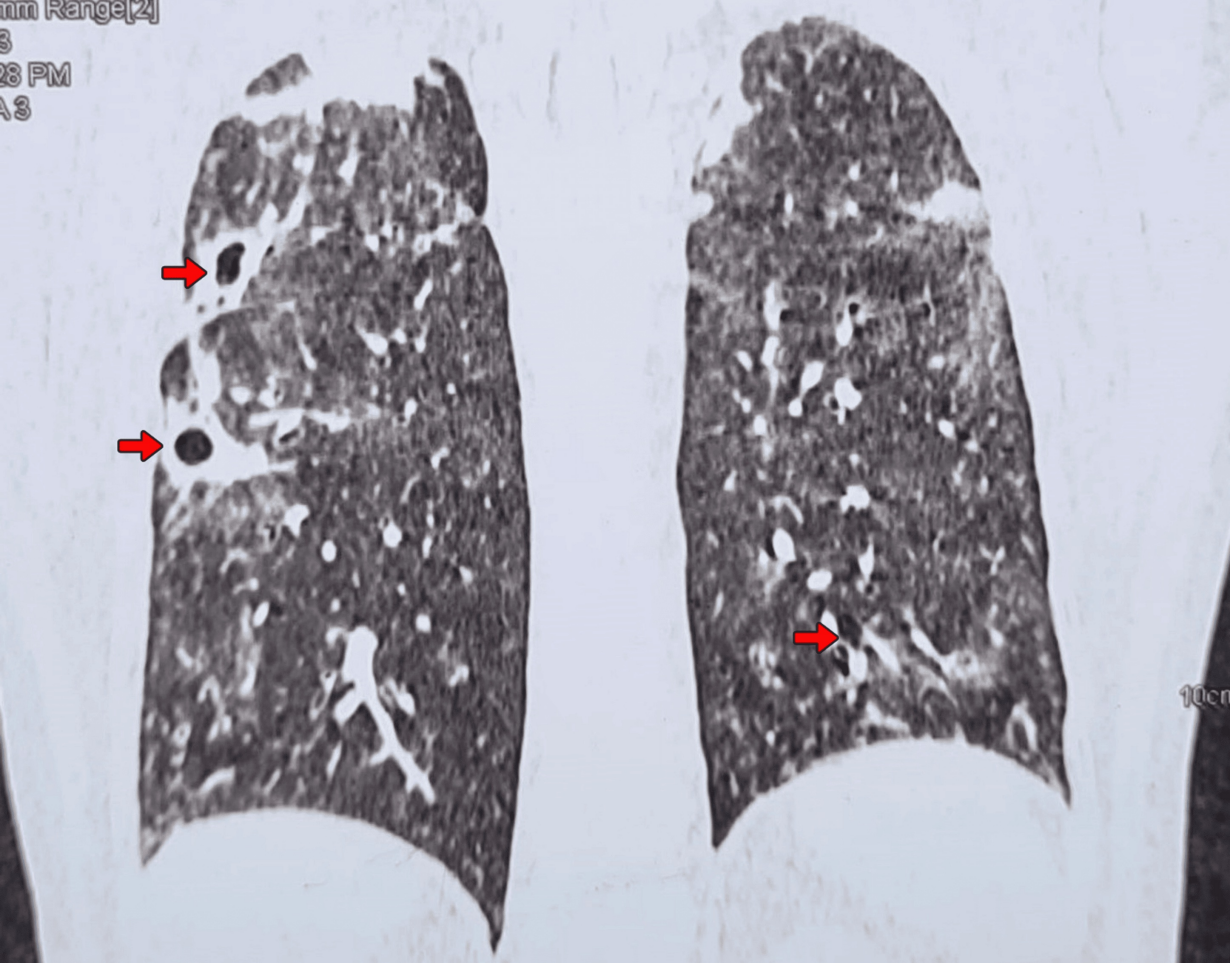
HRCT chest (coronal view) – cavitary lesions in upper lobes Coronal section of high-resolution computed tomography (HRCT) chest showing extensive bilateral upper lobe cavitations with surrounding ground-glass opacities (red arrows). These features support possible vasculitic or necrotizing pathology involving the pulmonary parenchyma.

The most common presenting symptoms were oliguria, edema, fatigue, and dyspnea. Blood pressure on admission was elevated in all patients (range: 140/90 mmHg to 180/110 mmHg), with hypertensive urgency observed in two cases. Notably, two patients (Cases 1 and 3) exhibited pulmonary involvement with cavitary lesions on HRCT chest (Figures 1, 2), although systemic vasculitic symptoms were absent in all cases.

Laboratory parameters

All patients exhibited anemia, with a calculated mean hemoglobin level of 6.98 g/dL, ranging between 5.6 and 8.7 g/dL. All patients demonstrated elevated white blood cell counts and raised inflammatory markers-CRP ranging from 68 to 134 mg/L and ESR from 82 to 108 mm/hr. Renal function was severely impaired across the cohort: mean serum creatinine was 7.03 mg/dL, and blood urea averaged 149 mg/dL. Electrolyte disturbances were common, including hyponatremia and mild hyperkalemia, with metabolic acidosis (low bicarbonate) observed in all cases (Table 1).

**Table 1:** Comparison of variables Comparison of laboratory findings and general considerations reveals homogeneity. NA^+^: sodium; K^+^: potassium; ESR: erythrocyte sedimentation rate; CRP: C-reactive protein; ANCA: anti-neutrophil cytoplasmic antibodies; ANA: antinuclear antibodies; dsDNA: double-stranded DNA

Parameter	Normal Range	Case 1	Case 2	Case 3	Case 4	Case 5	Case 6	Case 7
Age (Years)	–	56	43	57	63	62	58	47
Sex	–	Female	Female	Male	Female	Male	Female	Male
Hemoglobin (g/dL)	12–16(F), 13–17(M)	5.7	7.4	8.7	6.8	6.9	5.6	7.8
WBC (×10³/mm³)	4–11	15.3	13.8	14.2	15.6	14.8	16.2	13.9
Platelets (×10⁵/mm³)	1.5–4.0	2.2	2.5	2.1	2.0	2.3	2.4	2.1
Serum Creatinine (mg/dL)	0.6–1.2	6.5	4.8	7.2	9.3	8.1	7.9	5.4
Blood Urea (mg/dL)	15–45	145	132	158	170	162	147	135
Na^+^ (mEq/L)	135–145	133	135	130	132	134	132	132
K^+^ (mEq/L)	3.5–5.1	5.1	4.9	5.3	5.2	5.0	4.3	5.1
Bicarbonate (mmol/L)	22–28	15	18	14	12	13	13	16
ESR (mm/hr)	<20	94	82	101	108	97	100	85
CRP (mg/L)	<5	112	92	87	134	84	98	68
Urine Culture	No Growth	Klebsiella	E. coli	Sterile	Pseudomonas	Klebsiella	Proteus	E. coli
Chest Imaging	Normal	Cavities	Normal	Cavities	Normal	Normal	Infiltrates	Normal
ANCA, ANA, dsDNA	Negative	Negative	Negative	Negative	Negative	Negative	Negative	Negative
Complements (C3/C4)	Normal	Normal	Normal	Normal	Normal	Normal	Normal	Normal
Histopathology	–	80% crescents	70% crescents	85% crescents	90% crescents	80% crescents	85% crescents	75% crescents
Dialysis Sessions	–	11	9	13	7	10	6	12
Hospital Stay (days)	–	22	17	25	12	18	10	23
Outcome	–	Partial recovery	Partial recovery	Slow response	Death	Partial recovery	Death	Partial recovery

Immunologic workup

None of the patients demonstrated seropositivity for ANCA, including both p-ANCA and c-ANCA types. Additionally, tests for ANA, anti-dsDNA, and anti-GBM antibodies were also negative in all cases. Complement levels (C3 and C4) were within normal ranges in all patients, supporting the pauci-immune nature of glomerular involvement.

Urine and radiological findings

**Figure 3:**
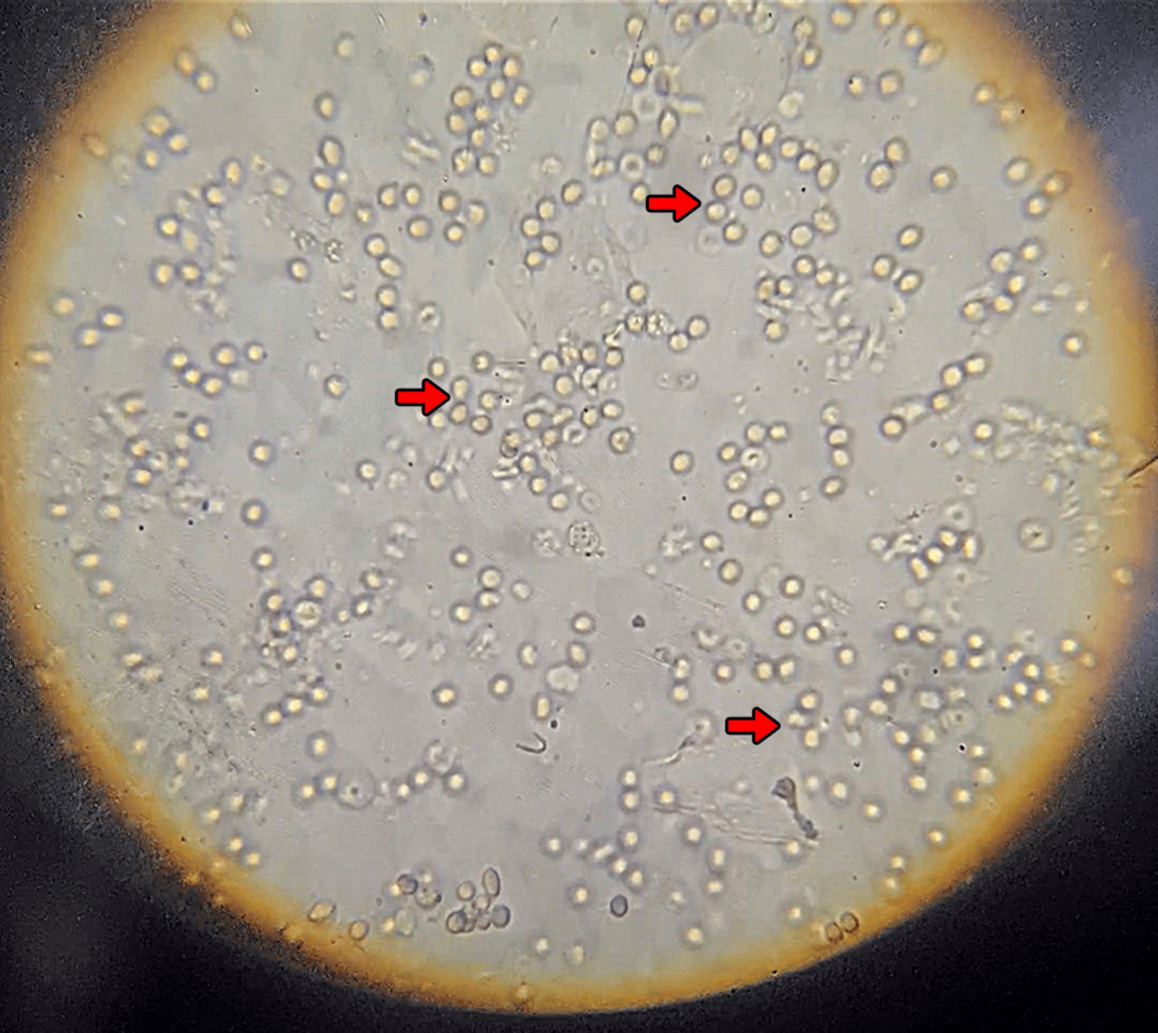
Urine microscopy – active urinary sediment Microscopic examination of an uncentrifuged urine sample shows numerous dysmorphic red blood cells (red arrows) and white blood cells. This finding is consistent with glomerular hematuria and active urinary sediment seen in crescentic glomerulonephritis.

Active urinary sediment was seen in all cases, with dysmorphic red cells (Figure 3) and granular casts. Urine culture was positive in five patients, with *Klebsiella, E. coli,* *Proteus*, and *Pseudomonas* species isolated, indicating secondary urinary tract infections. Chest imaging revealed pulmonary cavities in two cases and bilateral infiltrates in one case; four patients had normal chest findings.

Histopathology

**Figure 4:**
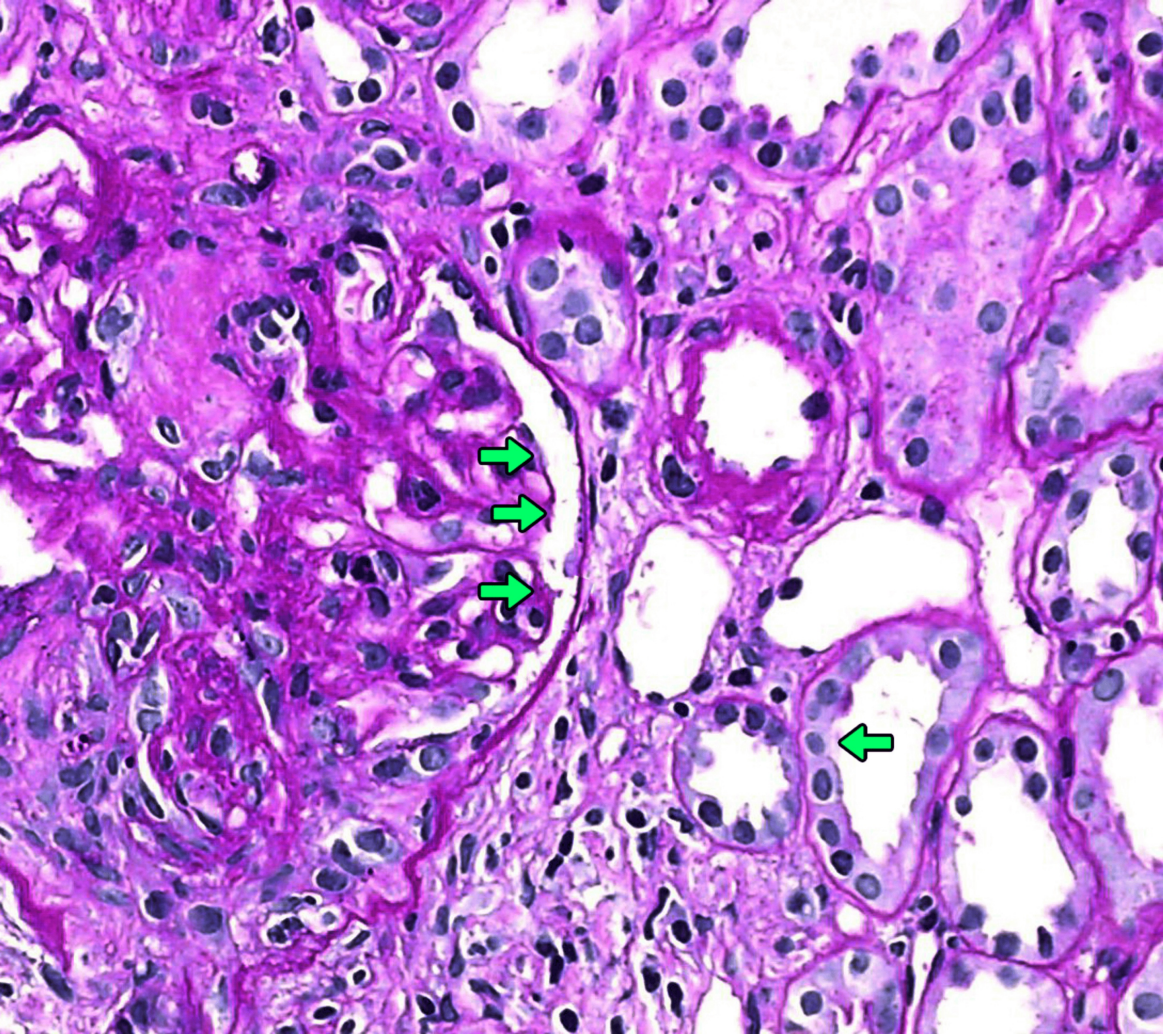
Renal biopsy – cellular crescents in glomerulus (PAS, 400x) Periodic acid-Schiff (PAS) stained section of renal tissue displaying a glomerulus with marked cellular crescent formation and disruption of Bowman’s capsule integrity (green arrows). No immune complex deposition noted. Histopathology confirms pauci-immune crescentic glomerulonephritis.

Renal biopsy findings were consistent with PICGN, with 70-90% glomeruli showing crescent formation. Immunofluorescence analysis revealed the absence of immune complex deposits across all biopsy specimens (Figure 4).

Treatment and outcomes

All seven patients received high-dose corticosteroids and cyclophosphamide, alongside supportive care. Dialysis was initiated in all cases, with sessions ranging from six to 13 (mean: 9.7 sessions per patient). The mean hospital stay per patient was 17.4 days (range: 10-25 days).

Outcomes varied

The seven patients exhibited a wide range of clinical outcomes, reflecting significant heterogeneity in disease progression and treatment response. Four individuals, specifically Cases 1, 2, 5, and 7, experienced partial recovery of renal function and were discharged without the need for ongoing dialysis support. Case 3 showed a slower, yet gradually improving renal trajectory, marked by persistent dysfunction despite clinical stabilization. In contrast, two patients, Cases 4 and 6, succumbed during their hospital stay. Both received immunosuppressive therapy and dialysis support, but their conditions deteriorated rapidly, highlighting the unpredictable and often severe nature of ANCA-negative PICGN.

Comparative observations

Several notable patterns emerged when comparing the clinical and laboratory profiles of the seven patients. Individuals who demonstrated poorer outcomes, particularly those who died, tended to have a greater percentage of glomerular crescents on biopsy (≥ 85%) coupled with more severe anemia, with baseline hemoglobin levels falling below 6.5 g/dL. Interestingly, while pulmonary involvement was detected in two patients via HRCT, its presence alone did not directly correlate with mortality. Instead, concurrent infections and significant metabolic disturbances, particularly severe acidosis reflected by low serum bicarbonate levels, were more consistently observed in the fatal cases. Conversely, patients who received early immunosuppressive therapy in conjunction with timely dialysis showed more favorable trends, suggesting that prompt intervention may be critical in altering the trajectory of this aggressive disease.

Statistical analysis

Given the limited sample size (n=7), statistical evaluation was primarily descriptive. Continuous variables such as age, hemoglobin, serum creatinine, urea, and inflammatory markers were reported as means with ranges. Categorical variables, including sex, urine culture results, chest imaging findings, dialysis requirement, and outcomes, were summarized using frequency and percentage distributions.

Across the seven cases reviewed, the average age of patients was 55 years, with ages ranging from 43 to 63 years. Hemoglobin levels were notably low, with a mean value of 6.98 g/dL, reflecting the presence of moderate to severe anemia across the cohort; values ranged between 5.6 and 8.7 g/dL. Renal function markers were consistently deranged at presentation, mean serum creatinine was 7.03 mg/dL (range: 4.8-9.3 mg/dL), and blood urea averaged 149 mg/dL, with individual values spanning from 132 to 170 mg/dL. Inflammatory markers were also markedly elevated, as evidenced by a mean CRP of 90.43 mg/L (range: 68-134 mg/L) and an average ESR of 95.3 mm/hour (range: 82-108 mm/hour). Patients underwent an average of 9.7 hemodialysis sessions, with the number ranging from six to 13 per patient, indicating a high level of renal support required during the acute phase. The mean duration of hospital stay was 17.4 days, with individual admissions lasting from 10 to 25 days.

Upon cross-analyzing clinical parameters with patient outcomes, several notable trends emerged. Both of the patients who succumbed to their illness exhibited crescentic involvement of 85% or more on renal biopsy, had evidence of significant metabolic acidosis with bicarbonate levels at or below 13 mmol/L, and required at least six sessions of hemodialysis during their hospital stay. Pulmonary manifestations such as cavitary lesions or infiltrates were identified in three of the seven patients; however, pulmonary involvement did not consistently predict mortality, as only one of these cases resulted in death. Among the five surviving patients, most had hemoglobin levels above 6.5 g/dL and initial serum creatinine levels below 8.5 mg/dL, suggesting that better baseline hematologic and renal parameters may be associated with improved short-term outcomes in ANCA-negative PICGN.

No inferential statistical tests (e.g., chi-square, t-tests) were performed due to small sample size and lack of a control group. Statistical calculations were performed using Microsoft Excel 2021 (Microsoft Corporation, Redmond, Washington, United States).

## Discussion

ANCA-negative PICGN is a diagnostically challenging and clinically aggressive renal disease due to its seronegative status and rapid progression [1,2]. In our case series, all seven patients presented with AKI, and most required hemodialysis. This observation is consistent with prior research by Chen and colleagues as well as Little et al., who noted that patients lacking detectable ANCA frequently present with more advanced renal dysfunction at the time of diagnosis compared to those with ANCA-positive disease [2,4].

Histologically, the hallmark of PICGN is the presence of extensive glomerular crescent formation with little or no immune deposits, as seen in our cohort with 70-90% crescent involvement [5]. This distinguishes it from other glomerulopathies such as lupus nephritis or IgA nephropathy, where immune complex deposition is a defining feature [5].

Pulmonary findings were observed in two patients, with HRCT revealing cavitary lesions, raising suspicion for granulomatosis with polyangiitis. However, the absence of ANCA and systemic features favored a renal-limited vasculitis, as supported by similar reports in the literature [6,13]. A rare instance of ANCA-negative pauci-immune glomerulonephritis with pulmonary-renal involvement was also described by Sethi et al., highlighting the spectrum of seronegative presentations [14].

Five of our patients also had positive urine cultures with Gram-negative organisms such as *E. coli, Klebsiella,* and *Proteus*. Prior studies suggest that infections might either act as a trigger for autoimmune activation or complicate the disease course, particularly in immunosuppressed individuals [7,15]. Additionally, drug-induced immune mechanisms have been recognized as potential contributors to pauci-immune glomerular injury, particularly in patients with no detectable ANCA, as described in medication-related cases [16].

All patients in our series were treated with corticosteroids and cyclophosphamide per institutional protocols. This is consistent with Kidney Disease Improving Global Outcomes (KDIGO) guidelines for managing crescentic GN, which recommend prompt immunosuppressive therapy irrespective of ANCA status [10]. Despite therapy, two patients succumbed, and only four achieved partial renal recovery, highlighting the high morbidity and mortality associated with ANCA-negative PICGN.

Liu et al. highlighted how acute kidney injury can drive systemic inflammatory responses, which may indirectly influence disease course and prognosis. Histopathologic evaluation, especially assessing the proportion of glomeruli with crescent formation, is a key determinant in forecasting prognosis in cases of pauci-immune rapidly progressive glomerulonephritis [9,17]

Recent advances suggest that novel biomarkers or molecular profiling may aid in diagnosing seronegative cases and guiding personalized therapy [6,18]. Until such tools are routinely available, high clinical suspicion, early biopsy, and aggressive immunosuppression remain the cornerstones of management.

Strengths and limitations

One of the notable strengths of this study lies in its contribution to the limited literature on ANCA-negative PICGN, particularly within the South Indian demographic. Each case included in this series was confirmed through renal biopsy, thereby enhancing diagnostic accuracy and reducing the risk of misclassification. The methodology adopted was comprehensive, encompassing clinical evaluation, laboratory parameters, imaging studies, microbiological cultures, and histopathological analysis, offering a detailed and multifaceted understanding of disease presentation and progression. Additionally, all patients were managed under a standardized treatment protocol within a single tertiary care center, which ensured uniformity in therapeutic interventions and outcome assessment. With seven cases presented, this report represents one of the more extensive series of ANCA-negative PICGN from this region, making it a valuable reference for clinicians encountering similar presentations.

However, the study is not without its limitations. Its retrospective nature introduces potential biases, including selection bias and gaps in clinical documentation, which may influence data interpretation. The small sample size limits statistical power and restricts the ability to draw broadly generalizable conclusions. Moreover, the absence of extended follow-up data hinders a comprehensive evaluation of long-term renal function and survival outcomes. Another constraint was the unavailability of advanced immunologic assays, which may have limited the detection of less common or novel antibodies that could be implicated in disease mechanisms. Finally, being a single-center experience, the applicability of findings across diverse populations and healthcare settings remains uncertain, underlining the need for larger, multicenter prospective studies to validate and expand upon these observations.

## Conclusions

Effective and prompt therapy is essential in managing ANCA-negative PICGN, a condition made more difficult to diagnose by the lack of conventional serological indicators. Despite their ANCA negativity, affected patients frequently demonstrate clinical features similar to ANCA-positive cases, including severe glomerular injury and the potential for rapid progression to end-stage renal disease. Our case series highlights the heterogeneous clinical presentation and reinforces the essential role of kidney biopsy in diagnosis. Initiating immunosuppressive therapy early, regardless of ANCA status, remains key to improving patient outcomes. In our cohort, five patients showed favorable responses, while two unfortunately succumbed, underscoring the importance of vigilance and early therapeutic intervention. There is a pressing need for continued research into the disease's underlying mechanisms and for the identification of novel biomarkers to enhance diagnostic accuracy and enable personalized treatment strategies for this rare but aggressive glomerulopathy.
